# Circ_0002984 promotes proliferation, migration and inflammatory cytokine secretion and inhibits apoptosis of rheumatoid arthritis fibroblast-like synoviocytes by inducing PCSK6 through miR-543

**DOI:** 10.1186/s13018-023-03823-4

**Published:** 2023-05-06

**Authors:** Jian-zuo Lu, Jie Yang, Sheng-tuo Zhou, Kai-luo Xie

**Affiliations:** Department of Orthopedics, The People’s Hospital of Wenzhou City, No. 57, Canghou Lane, Wenzhou, 325000 China

**Keywords:** Rheumatoid arthritis, Circ_0002984, MiR-543, PCSK6, Rheumatoid arthritis fibroblast-like synoviocytes

## Abstract

**Background:**

Rheumatoid arthritis (RA) is inflammatory arthritic disease, and circular RNA is involved in RA development. The aim of the present work is to analyze the role of circ_0002984 in the process of RA fibroblast-like synoviocytes (RAFLSs) and the underlying mechanism.

**Methods:**

Circ_0002984, miR-543, and proprotein convertase subtilisin/kexin type 6 (PCSK6) expression levels were analyzed by quantitative real-time polymerase chain reaction or western blotting. Cell proliferation, migration, inflammatory response, and apoptosis were investigated through 5-Ethynyl-2′-deoxyuridine assay, wound-healing assay, enzyme-linked immunosorbent assay, and flow cytometry analysis. Dual-luciferase reporter assay and RNA immunoprecipitation assay were performed to assess the binding relationship.

**Results:**

Circ_0002984 and PCSK6 expression were increased, while miR-543 expression was decreased in the synovial tissues of RA patients and RAFLSs. Circ_0002984 introduction facilitated RAFLS cell proliferation, migration and inflammatory response and repressed apoptosis, but circ_0002984 knockdown had an opposite role. Circ_0002984 targeted miR-543, and PCSK6 was targeted by miR-543. MiR-543 downregulation or PCSK6 overexpression restored the effects of circ_0002984 interference on RAFLS phenotypes.

**Conclusion:**

Circ_0002984 promoted RAFLS proliferation, migration and inflammatory cytokine secretion and inhibited apoptosis by binding to miR-543 to induce PCSK6 production, providing a potential target for RA therapy.

## Introduction

Rheumatoid arthritis (RA) is a chronic and debilitating systemic autoimmune disease with concomitant disability and impacts around 1% of the population, accompanied by systemic immune and inflammatory manifestations [1]. Although much progress has been achieved in RA therapy such as anti-rheumatic drug strategies, most patients still experience pain and overall remission rate is still unsatisfactory [2]. Fibroblast-like synoviocytes (FLSs), type B synoviocytes, are a kind of chiefly constituent cells in RA and their phenotypes can be altered during the pathogenesis of RA [3]. Thus, identification of FLS function is necessary to obtain new insights into the mechanism responsible for RA development and to develop innovative therapeutic strategies.

Research data have indicated that circular RNA (circRNA) plays a vital role in autoimmune diseases, including RA [4, 5]. CircRNA forms by the head-to-tail splicing event and its biological characteristics include high stability, wide expression, cell-or tissue-specific expression pattern and aberrant expression in diseases [6]. CircRNA can act biological functions by microRNA (miRNA) response elements, which promote the binding of circRNA for miRNA [7]. Some studies have shed light on circRNA function in RA development. For example, circ_0088194 promoted RAFLS migration and invasion through the miR-766-3p in vitro [8]. CircRNA fragile mental retardation 2 targeted the miR-650/2′,3′-cyclic nucleotide 3′-phosphodiesterase (CNP) pathway to increase RAFLS proliferation and inflammatory response, as revealed by an in vitro cell model assay [9]. Circ_0002984 forms from the Rho GTPase Activating Protein 32 (ARHGAP32) gene through back-splicing and has a high expression in the peripheral blood mononuclear cells from RA patients, as analyzed through the GSE189338 dataset in the preliminary experiment; however, no study has been performed to analyze circ_0002984 function in RA development.

MiRNAs are small RNAs of 22–24 nucleotides and can target their complementary mRNAs, further inhibiting or silencing genes at post-transcriptional levels. MiRNAs are related to different regulatory pathways and thus participate in disease development [10, 11]. At present, some reports have explained their vital functions in immune and inflammatory diseases. Many miRNAs are abnormally expressed in body fluids of RA patients, and their expression also is associated with RA stage [12]. Serum miRNA level can be utilized to predict response to RA therapy [12]. With further research, it is found that miRNAs regulate the biological behaviors of RA, such as synovial inflammation, osteoclastogenesis, joint destruction and knee-joint homeostasis [13]. As reported, circRNA can work as miRNA sponge to reduce miRNA expression level, thus modulating miRNA target gene expression [14]. Based on the above evidence, starbase online database was used to predict circ_0002984-binding miRNA and the miRNA-binding mRNA. As a result of identification, we assembled the circ_0002984/miR-543/PCSK6 axis to reveal the mechanism of circ_0002984 regulating RA development using MH7A cells.

Herein, we investigated the effects of circ_0002984 on MH7A cell proliferation, migration, inflammatory response and apoptosis and determined whether the miR-543/PCSK6 axis was involved in circ_0002984-regulated MH7A cell phenotypes.

## Materials and methods

### Synovial tissue collection

Thirty-seven RA patients suffered from joint surgery and 37 patients with joint trauma provided synovial tissues in the People’s Hospital of Wenzhou City. The control group not included patients with autoimmune or infectious diseases. The issues were stored at − 80 °C. All participants signed the written informed consent. The Ethics Committee of the People’s Hospital of Wenzhou City approved the study.

### Cell culture

Rheumatoid arthritis fibroblast-like synoviocytes (MH7A) and normal fibroblast-like synoviocytes (FLS) were purchased from Otwo Biotech (Shenzhen, China) and cultured in RPMI-1640 medium added with 10% fetal bovine serum at 37 °C.

### Cell transfection

Small interfering RNA of candidate circRNA (si-hsa_circ_0002984, 5′-CAGATTGCTTGTCAGGCAAGA-3′), miR-543 mimics (5′-AAACAUUCGCGGUGCACUUCUU-3′), miR-543 inhibitors (5′-AAGAAGUGCACCGCGAAUGUUU-3′) and the matched controls (si-NC and NC mimic) were provided by GenePharma (Shanghai, China). Circ_0002984 and PCSK6 overexpression plasmids and controls were built in Songon Biotech (Shanghai, China). MH7A cells were transfected with the above plasmids and oligonucleotides in line with the standard instructions of Lipofectamine 2000 (Invitrogen, Carlsbad, CA, USA) and maintained in 24-well plates. Before transfection, MH7A cells were cultured in serum-free medium for a whole day, and the complexes were added to each well. The cells were collected at the defined time.

### Quantitative real-time PCR (qRT-PCR)

RNA from synovial tissues and fibroblast-like synoviocytes was isolated using Trizol (Thermo Fisher, Waltham, MA, USA). Nucleocytoplasmic RNA of MH7A cells was isolated based on the guidebook of nucleocytoplasmic RNA separation reagents (Norgen Biotek, Thorold, Canada). RNA was used for reverse transcription with miRNA or mRNA reverse transcriptase kits (Thermo Fisher). Then, cDNA was reacted on a qRT-PCR thermocycler (Thermo Fisher) with SYBR (Tsingke, Shanghai, China). RNA expression was analyzed through the 2^−∆∆Ct^ method. The primer pairs are shown in Table 1. All primers were synthesized in Tsingke Biotech.

**Table 1:** Primers sequences used for PCR

Name		Primers for PCR (5′–3′)
hsa_circ_0002984	Forward	CGGCAGCATACAGCTTTCAC
	Reverse	GCGCCTCTTGCCTGACA
miR-543	Forward	GGTCGAAAACATTCGCGGTG
	Reverse	TCCGAGGTATTCGCACTGGA
PCSK6	Forward	ACGACGTGAACGGCAATGATT
	Reverse	TTCTCCCGCACAACGAGTG
MMP3	Forward	TGAGGACACCAGCATGAACC
	Reverse	ACTTCGGGATGCCAGGAAAG
GAPDH	Forward	GGAGCGAGATCCCTCCAAAAT
	Reverse	GGCTGTTGTCATACTTCTCATGG
U6	Forward	CTCGCTTCGGCAGCACA
	Reverse	AACGCTTCACGAATTTGCGT

### RNase R treatment

RNase R digestion reaction was conducted as previously shown [15]. RNA was then subjected to qRT-PCR analysis to determine circ_0002984 and GAPDH expression.

### Cell viability assay

According to the guidebook, cell viability was analyzed using Cell Counting Kit-8 (CCK-8; Dojindo, Shanghai, China). 200 μL of MH7A cells was seeded in 96-well plates. After 48 h of transfection, CCK-8 reagent was added to each well for 4 h. The absorbance was detected using a spectrophotometer.

### Cell proliferation assay

5-Ethynyl-2′-deoxyuridine (EdU) assay was performed to evaluate cell proliferation. After 24 h of treatment, MH7A cells were cultured in 96-well plates for 24 h. Then, the assay was conducted as per the instruction of EdU apollo 567in vitro kit (Solarbio, Beijing, China). Finally, cell proliferation rate was determined under fluorescence microscope.

### Wound-healing assay

The assay was performed based on the reported method [16]. MH7A cells were subjected to various transfections and cultured for 24 h. Wounds were created using plastic pipette tips, and the cells were maintained in serum-free RPMI-1640 medium at 37 °C. Cell debris was removed and wound-closing process was analyzed by determining the width of the scratch gap at 0 and 24 h under a microscope.

### Enzyme-linked immunosorbent assay (ELISA)

MH7A cells with various transfections were maintained in 24-well plates for 48 h. The cell supernatant was harvested and TNF-α and IL-1β levels in the supernatant were analyzed using commercial kits (#PT518 and #PI305; Beyotime, Shanghai, China) according to manufacturer’s direction.

### Apoptosis assay

Commercial cell apoptosis detection kit (MultiSciences Biotech, Hangzhou, China) was used for this assay. In brief, MH7A cells were fixed in 70% ethanol and stained with PI/Annexin V-FITC in the dark. A flow cytometer with FlowJo software was used to analyze cell apoptosis.

### Western blotting analysis

Radioimmunoprecipitation assay lysis buffer (Beyotime) was used to extract protein from synovial tissues and fibroblast-like synoviocytes according to the manufacturer's protocols. Protein samples were loaded onto SurePAGE gels (Thermo Fisher) and transferred onto polyvinylidene difluoride membrane using XCell II Blot Module (Thermo Fisher) prior to blocking with defatted dry milk. The membranes were incubated with BCL-2 antibody (#K003505P; 1:1000; Solarbio), Bax antibody (#K008076P; 1:1000; Solarbio), PCSK6 antibody (#PA5-68543; 1:400; Thermo Fisher) and GAPDH antibody (#K106389P; 1:5000; Solarbio). Membranes were subsequently incubated with anti-rabbit immunoglobulin G antibodies (Solarbio). Finally, enhanced chemiluminescence detection system was used to visualize protein bands.

### RNA immunoprecipitation (RIP)

As instructed [17], Sepharose beads (Bio-Rad, Hercules, CA, USA) were first incubated with AGO2 antibody (Abcam, Cambridge, MA, USA) or IgG antibody (Abcam). MH7A cells were disrupted in lysis buffer, and then lysates were subjected to incubation with pre-coated Sepharose beads for 3 h. After centrifuging samples at 12,000 rpm for 12 min, qRT-PCR was implemented to measure circ_0002984, miR-543 and PCSK6 expression.

### Dual-luciferase reporter assay

Starbase online database was used to predict the binding sites of circ_0002984 for miR-543 and miR-543 for PCSK6. The partial sequence in circ_0002984 or PCSK6 3′UTR that contained miR-543-binding sites along with the mutant sequence in circ_0002984 or PCSK6 3′UTR that contained the mutant sites were inserted into pmirGLO vector to generate WT-hsa_circ_0002984, MUT-hsa_circ_0002984, WT-PCSK6 3′UTR and MUT-PCSK6 3′UTR. Site-directed mutagenesis was performed in GenScript Biotech (Nanjing, China). Then, MH7A cells transfected with the above plasmids, miR-543 mimics or NC mimics were collected after 48 h for luciferase activity analysis in line with the guidebook of Dual-Lucy Assay Kit (Solarbio).

### Statistical analysis

Data were analyzed using GraphPad Prism and expressed as means ± standard deviations (SD). Significant differences were compared with Wilcoxon signed-rank test, Student’s *t*-tests, or one-way analysis of variance. *P* < 0.05 indicated statistical significance.

## Results

### Circ_0002984 expression was upregulated in the synovial tissues of RA patients

**Fig. 1:**
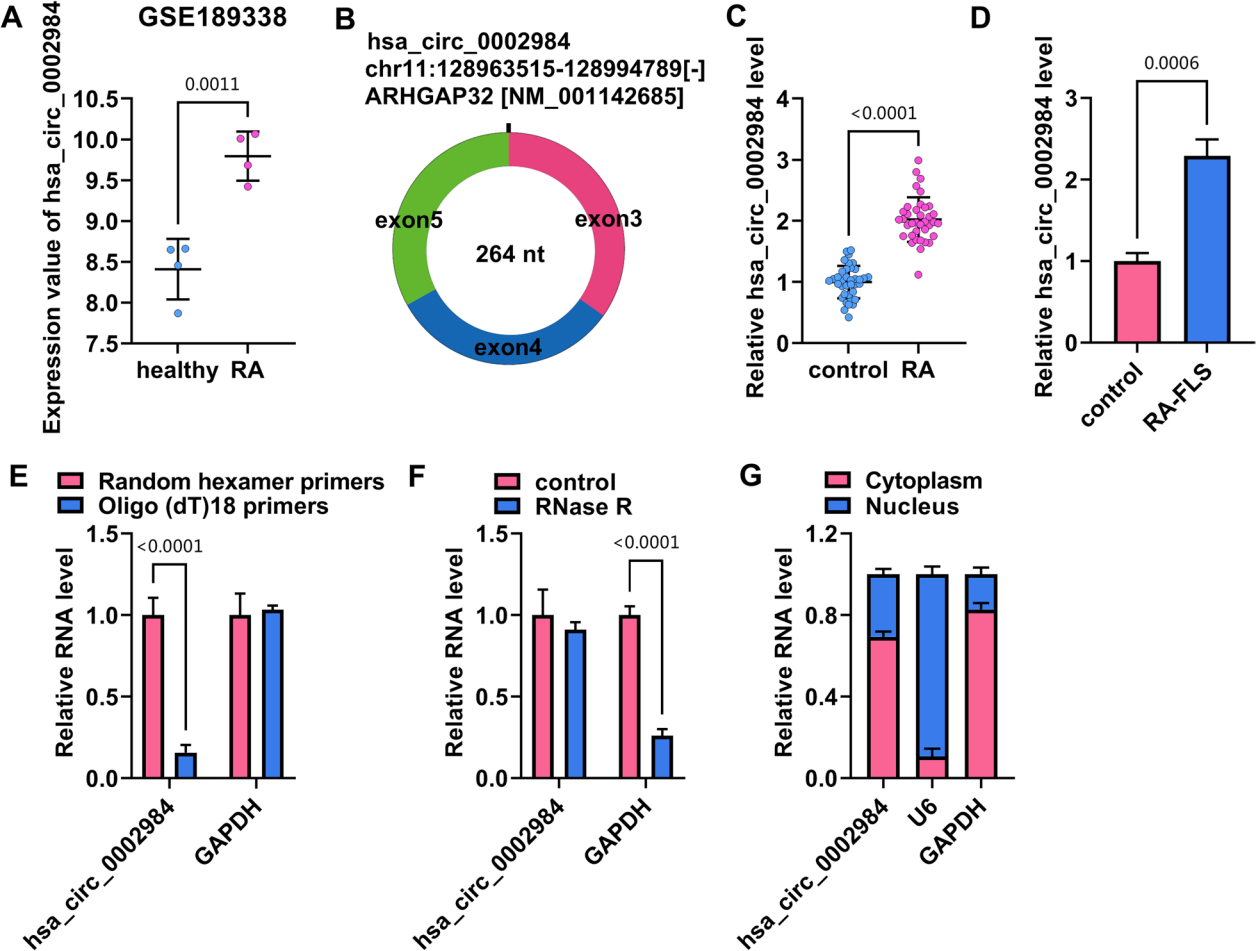
Circ_0002984 expression in the synovial tissues of RA patients. **A** Circ_0002984 expression was first analyzed in peripheral blood mononuclear cells from RA patients through GSE189338 dataset. **B** The schematic illustration showing the generation of circ_0002984. **C** Circ_0002984 expression was detected by qRT-PCR in the synovial tissues from RA patients and healthy controls. **D** Circ_0002984 expression was checked by qRT-PCR in RA-FLS (MH7A cells) and healthy FLS. **E** and **F** The circular structure of circ_0002984 was identified using oligo (dT) 18 primers, random hexamer primers and RNase R. **G** Nucleocytoplasmic separation assay was used to demonstrate that circ_0002984 was mainly located in the cytoplasm

Circ_0002984 expression was first analyzed in peripheral blood mononuclear cells from RA patients through GSE189338 dataset, and the result showed that circ_0002984 expression was upregulated in the cells (Fig. 1A). The Fig. 1B showed the generation of circ_0002984. Subsequently, qRT-PCR was used to check circ_0002984 expression in the synovial tissues from RA patients and healthy controls. As shown in Fig. 1C, circ_0002984 expression was significantly increased in the synovial tissues from RA patients when compared with controls. Consistently, circ_0002984 expression was higher in RA-FLS (MH7A cells) than in healthy FLS (Fig. 1D). Then, we confirmed the circular structure of circ_0002984 using oligo(dT)18 primers, random hexamer primers and RNase R. For example, circ_0002984 could be significantly amplified using random hexamer primers rather than oligo(dT)18 primers (Fig. 1E). Moreover, circ_0002984 was resistant to RNase R digestion (Fig. 1F). Further, the result showed that circ_0002984 was mainly expressed in the cytoplasm of MH7A cells (Fig. 1G). The above data suggested that circ_0002984 might participate in RA development.

### Circ_0002984 promoted MH7A cell proliferation, migration and inflammatory cytokine secretion and inhibited cell apoptosis

**Fig. 2:**
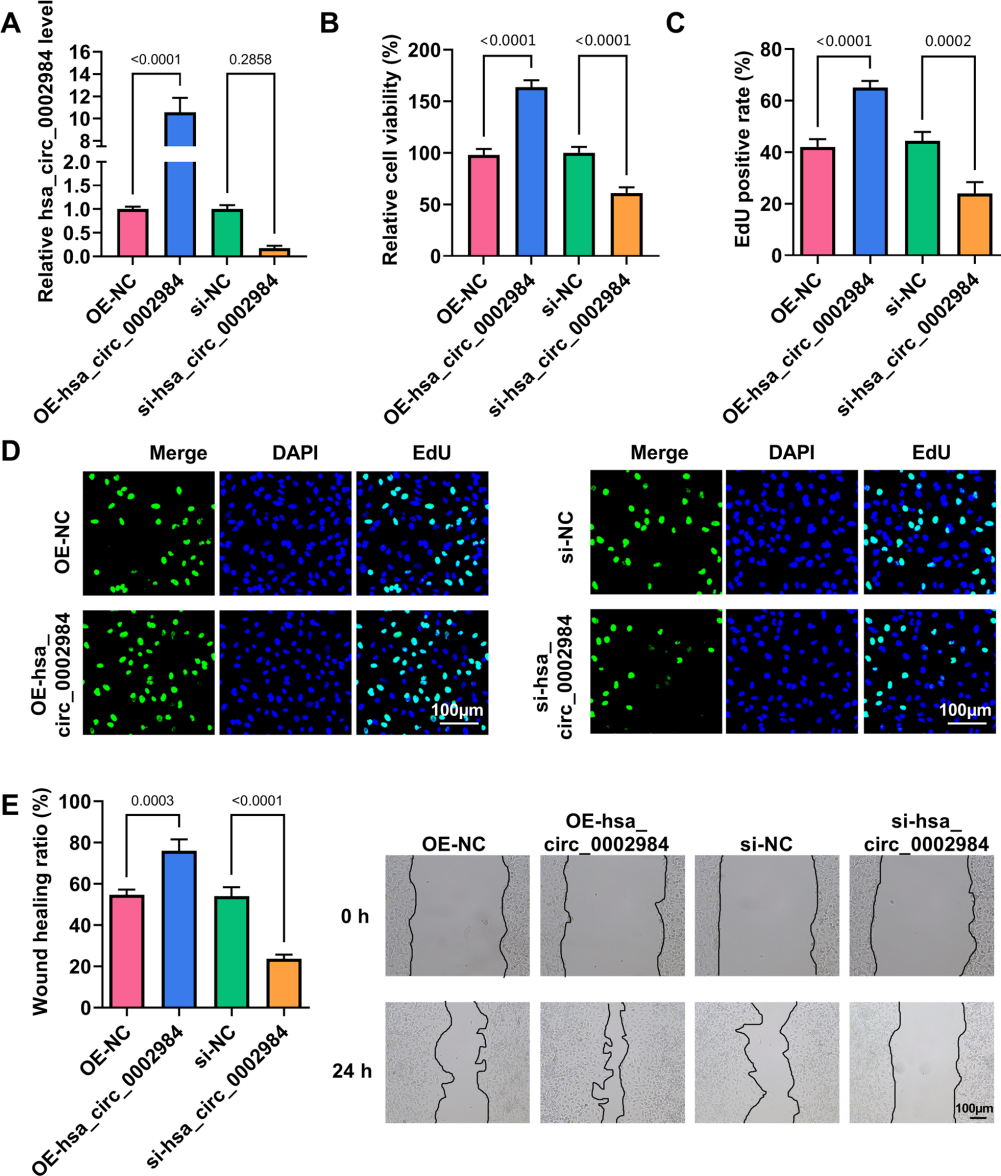
Circ_0002984 promoted MH7A cell proliferation and migration. MH7A cells were transfected with OE-hsa_circ_0002984, OE-NC, si-hsa_circ_0002984 or si-NC, and circ_0002984 expression was analyzed by qRT-PCR (**A**), cell viability by CCK-8 assay (**B**), cell proliferation by EdU assay (**C**, **D**), and cell migration by wound-healing assay (**E**)

**Fig. 3:**
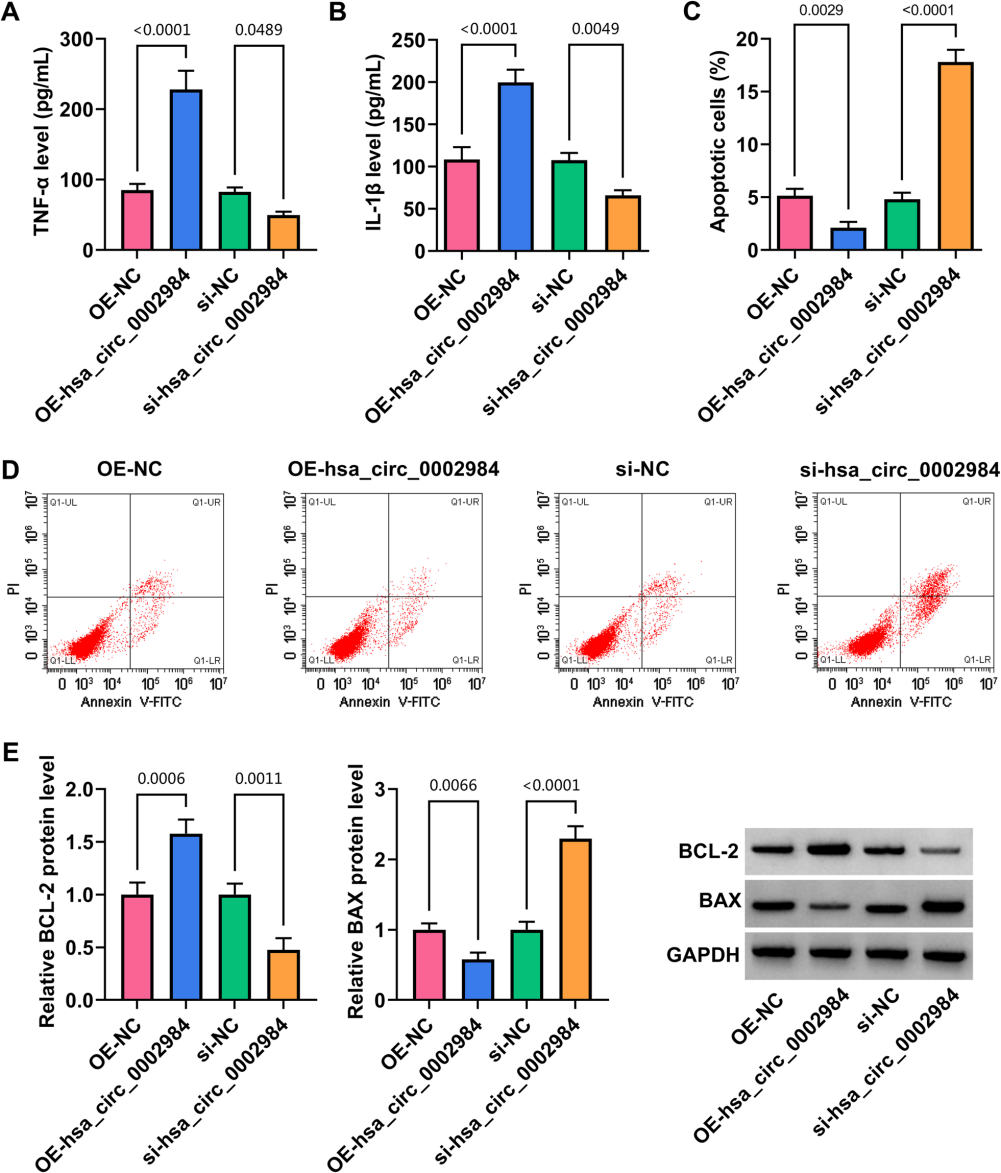
Circ_0002984 increased inflammatory cytokine secretion and decreased cell apoptosis. MH7A cells were transfected with OE-hsa_circ_0002984, OE-NC, si-hsa_circ_0002984 or si-NC, and TNF-α and IL-1β secretion were detected by ELISA (**A**, **B**), cell apoptotic rate by flow cytometry analysis (**C**, **D**) and BCL-2 and BAX protein expression by western blotting assay (**E**)

The study then analyzed the effects of circ_0002984 on MH7A cell processes. As shown in Fig. 2A, the efficiency of circ_0002984 overexpression and knockdown was high. Subsequently, ectopic circ_0002984 expression increased MH7A cell viability, proliferation and migration, but circ_0002984 silencing had the opposite effects (Fig. 2B–E). Moreover, circ_0002984 overexpression induced the secretion of TNF-α and IL-1β, whereas circ_0002984 depletion repressed TNF-α and IL-1β secretion (Fig. 3A, B). Further, the transfection of circ_0002984 overexpression plasmid inhibited MH7A cell apoptosis, accompanied by an increase of BCL-2 protein expression and a decrease of BAX protein expression; however, the transfection with si-circ_0002984 had the opposite effects (Fig. 3C–E). These findings demonstrated that circ_0002984 had the potency to promote MH7A cell proliferation, migration and inflammatory cytokine secretion and to inhibit apoptosis.

### Circ_0002984 bound to miR-543 in MH7A cells

**Fig. 4:**
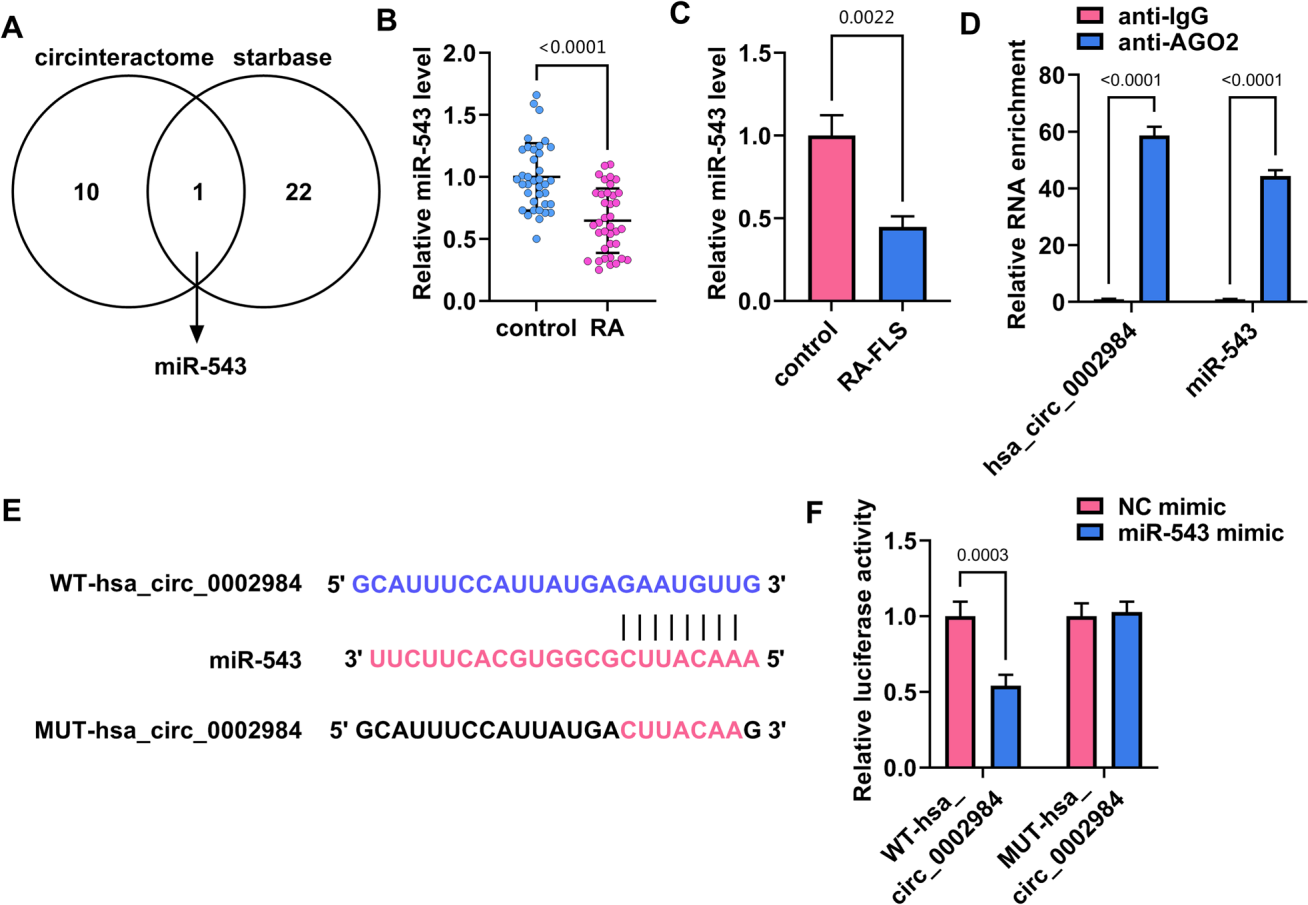
Circ_0002984 bound to miR-543 in MH7A cells. **A** The schematic illustration showing the miRNAs with circ_0002984 binding sites. **B** MiR-543 expression was detected by qRT-PCR in the synovial tissues from RA patients and healthy controls. **C** MiR-543 expression was checked by qRT-PCR in RA-FLS (MH7A cells) and healthy FLS. **D** RIP assay was performed to identify the association of circ_0002984 with miR-543. **E** The binding sites of circ_0002984 for miR-543. **F** Dual-luciferase reporter assay was conducted to determine the binding relationship of circ_0002984 and miR-543

Circ_0002984-associated miRNAs were predicted through circinteractome and starbase online databases. We found that only miR-543 had the binding sites of circ_0002984 after overlapping the prediction results, as shown in Fig. 4A. Subsequently, qRT-PCR analysis showed that miR-543 expression was significantly downregulated in the synovial tissues from RA patients when compared with the controls (Fig. 4B). Moreover, miR-543 expression was lower in RA-FLS (MH7A cells) than in healthy FLS (Fig. 4C). As shown in Fig. 4D, circ_0002984 and miR-543 were dramatically enriched in the anti-AGO2 group rather than anti-IgG group. The above results indicated that miR-543 might be a target miRNA of circ_0002984. The binding sites of circ_0002984 for miR-543 were shown in Fig. 4E. Further, dual-luciferase reporter assay was used to identify the possible association of circ_0002984 with miR-543. As presented in Fig. 4F, the co-transfection of miR-543 mimics with WT-hsa_circ_0002984 significantly inhibited the luciferase activity of MH7A cells, but the co-transfection of miR-543 mimics with MUT-hsa_circ_0002984 did not. Therefore, circ_0002984 bound to miR-543 in MH7A cells.

### MiR-543 targeted PCSK6 in MH7A cells

**Fig. 5:**
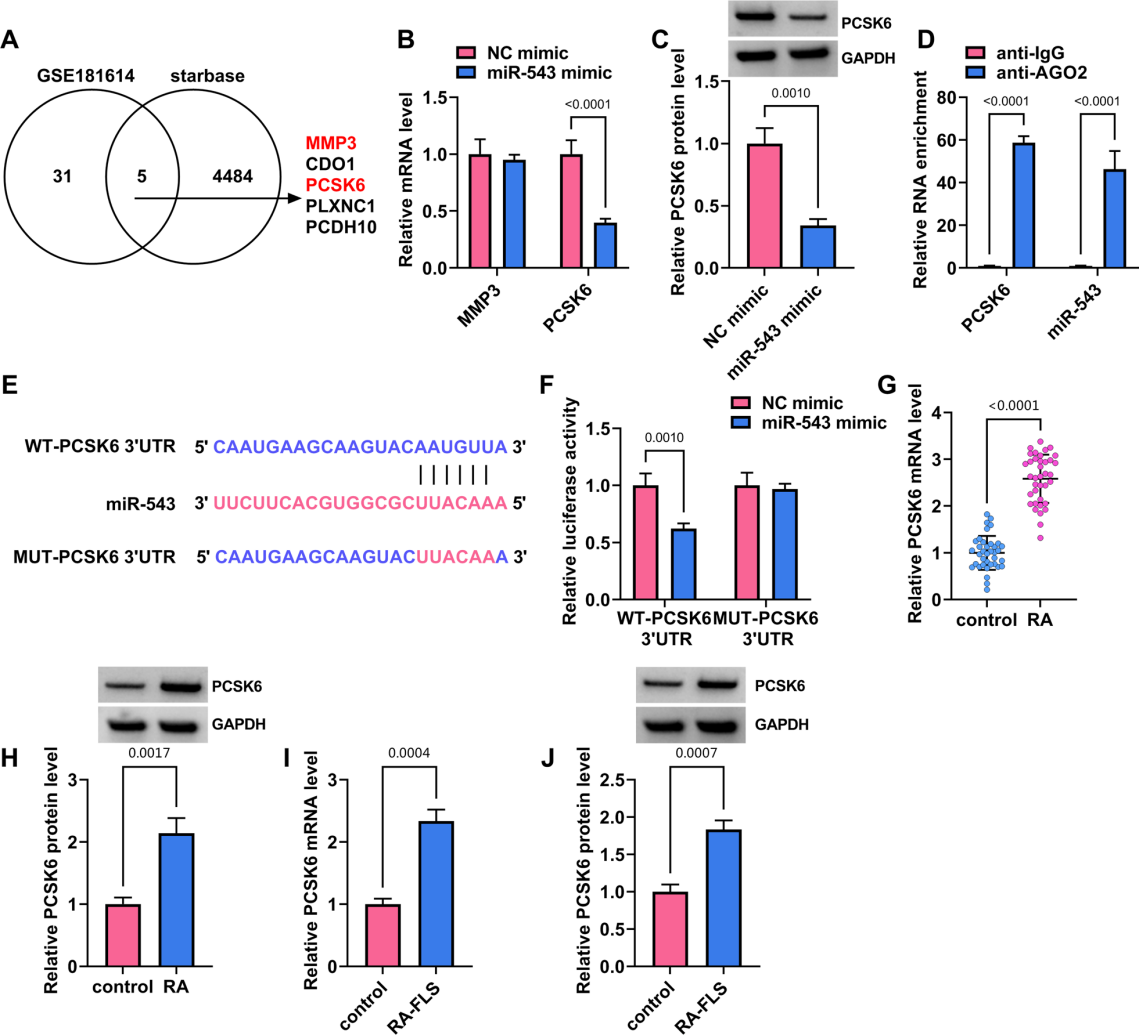
MiR-543 bound to PCSK6 in MH7A cells. **A** Starbase online database and GSE181614 dataset were used to predict mRNA with miR-543-binding sites. **B** The effects of miR-543 introduction on MMP3 and PCSK6 expression were determined by qRT-PCR in MH7A cells. **C** The effect of miR-543 mimics on PCSK6 protein expression was analyzed by western blotting assay. **D** RIP assay was used to identify the association of miR-543 with PCSK6. **E** The schematic illustration showed the binding sites of miR-543 for PCSK6. **F** Dual-luciferase reporter assay was performed to demonstrate that miR-543 bound to PCSK6. **G**–**J** PCSK6 expression was analyzed by qRT-PCR and western blotting in the synovial tissues from RA patients and healthy controls, RA-FLS (MH7A cells) and healthy FLS

The study continued to predict miR-543-associated mRNAs using starbase online database and GSE181614 dataset. mRNAs that were significantly upregulated in the FLS isolated from RA patients (fold change > 10, *p* < 0.05) were chosen for prediction. As shown in Fig. 5A, we found that 5 mRNAs (MMP3, CDO1, PCSK6, PLXNC1 and PCDH10) contained miR-543-binding sites after overlapping the prediction results. Among these mRNAs, MMP3 and PCSK6 were chosen for qRT-PCR analysis due to their important roles in RA progression. The results showed that miR-543 mimics inhibited PCSK6 expression but not MMP3 expression (Fig. 5B). Also, miR-543 mimics could repress PCSK6 protein expression (Fig. 5C). AGO2 antibody could dramatically enrich PCSK6 and miR-543, whereas IgG antibody did not (Fig. 5D). Thus, PCSK6 was selected as a potential target mRNA of miR-543. The binding sites of miR-543 for PCSK6 were shown in Fig. 5E. Dual-luciferase reporter assay showed that miR-543 introduction inhibited the luciferase activity of WT-PCSK6 3′UTR but not that of the luciferase activity of MUT-PCSK6 3′UTR (Fig. 5F). Further, we observed a high expression of PCSK6 in the synovial tissues from RA patients as well as RA-FLS (MH7A cells) (Fig. 5G–J). Therefore, miR-543 bound to PCSK6 in MH7A cells.

### Circ_0002984 depletion inhibited MH7A cell processes by regulating miR-543 and PCSK6

**Fig. 6:**
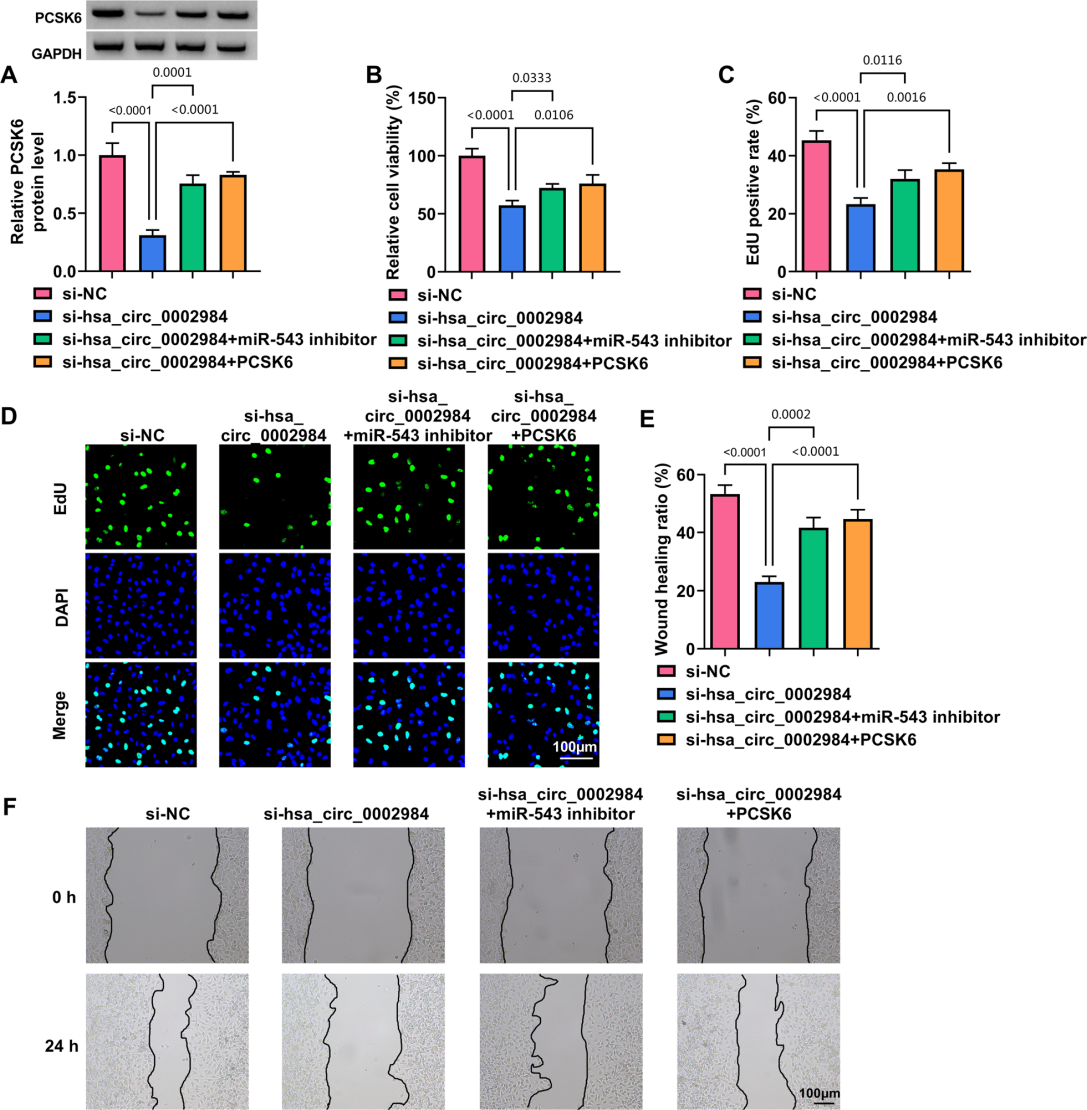
Circ_0002984 modulated MH7A cell proliferation and migration through miR-543 and PCSK6. MH7A cells were divided into si-NC group, si-hsa-circ_0002984 group, si-hsa-circ_0002984 + miR-543 inhibitor group and si-hsa-circ_0002984 + PCSK6 group, and PCSK6 protein expression was analyzed by Western blot (**A**), cell viability by CCK-8 assay (**B**), cell proliferation by EdU assay (**C**, **D**), and cell migration by wound-healing assay (**E**, **F**)

**Fig. 7:**
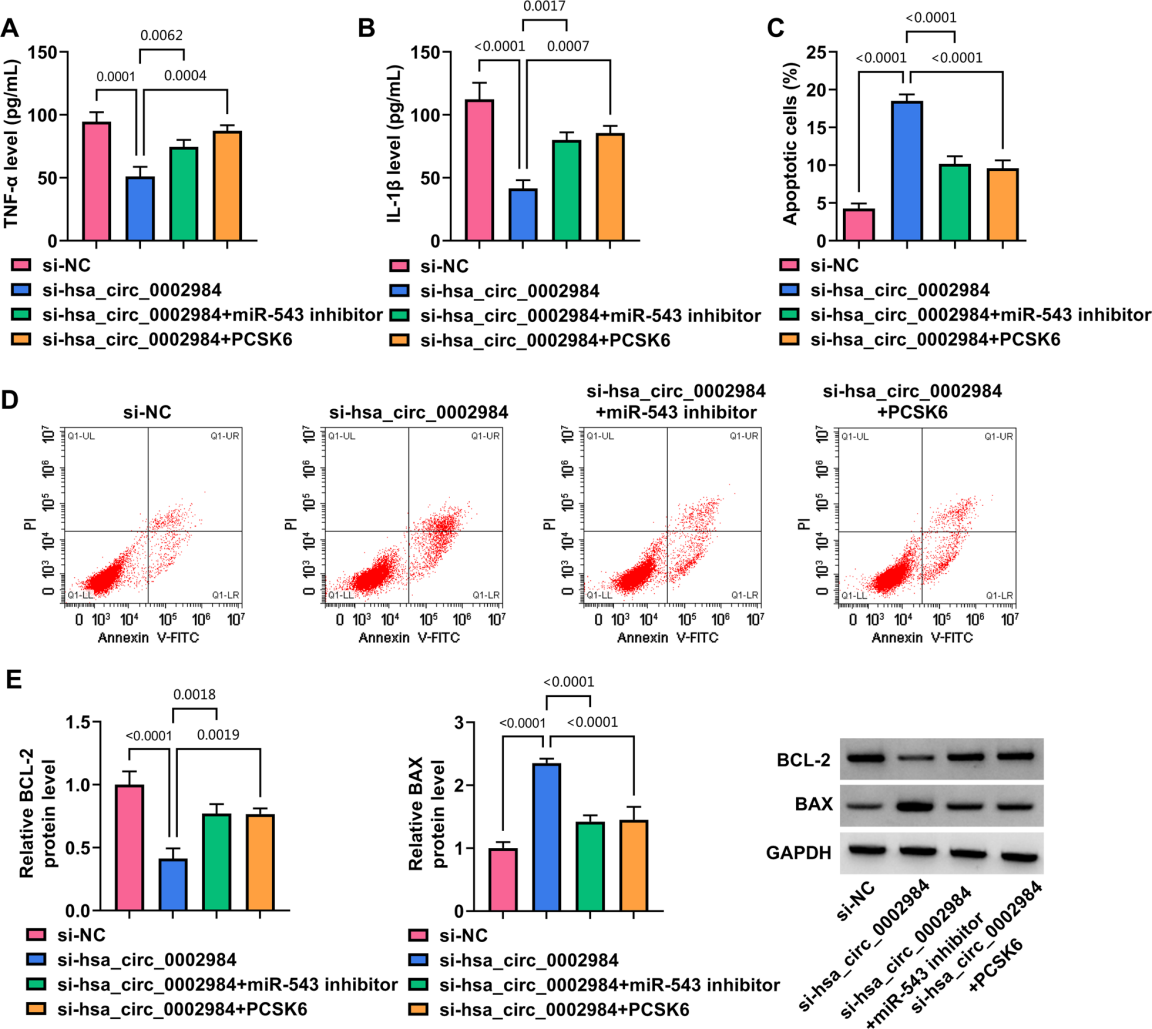
Circ_0002984 modulated inflammatory cytokine secretion and cell apoptosis through miR-543 and PCSK6. MH7A cells were divided into si-NC group, si-hsa-circ_0002984 group, si-hsa-circ_0002984 + miR-543 inhibitor group and si-hsa-circ_0002984 + PCSK6 group, and TNF-α and IL-1β secretion were detected by ELISA (**A**, **B**), cell apoptotic rate by flow cytometry analysis (**C**, **D**) and BCL-2 and BAX protein expression by western blotting assay (**E**)

Based on the above results, we analyzed whether miR-543 and PCSK6 participated in the regulation of circ_0002984 in MH7A cell phenotypes. The results first showed that circ_0002984 knockdown inhibited PCSK6 production, whereas the effect was attenuated after miR-543 depletion or PCSK6 overexpression (Fig. 6A). Subsequently, the decreased expression of circ_0002984 led to inhibition in cell viability, cell proliferation and cell migration, but these effects were rescued after miR-543 depletion or PCSK6 introduction (Fig. 6B–F). As shown in Fig. 7A–D, circ_0002984 silencing inhibited TNF-α and IL-1β secretion and induced cell apoptosis, but miR-543 inhibitors or PCSK6 overexpression counteracted these effects. Further, circ_0002984 knockdown decreased BCL-2 expression and increased BAX expression; however, these effects were restored by inhibiting miR-543 expression or promoting PCSK6 production (Fig. 7E). Thus, circ_0002984 modulated MH7A cell phenotypes through miR-543 and PCSK6.

## Discussion

RA is a chronic inflammatory disorder and influences joint tissues, bone, and cartilage. At present, mechanistic studies on RA are lacking and the pathogenesis of the disease is still blurry. Much evidence suggests that RA development, diagnosis and prognosis involve circRNAs. In the present work, we analyzed the function of a novel circRNA, circ_0002984, in RAFLS proliferation, migration, inflammatory response and apoptosis, and the detailed mechanism. The results showed that circ_0002984 silencing had the potential to hinder RA development and the molecular mechanism involved the circ_0002984/miR-543/PCSK6 axis.

This work confirmed the high expression of circ_0002984 in synovial tissues from RA patients and RAFLSs. Circ_0002984 had a circular structure and was resistant to RNase R digestion. In addition, circ_0002984 was cytoplasmic circRNA. Functional assays showed that circ_0002984 could promote RAFLS proliferation, migration and inflammatory cytokine secretion. On the contrary, the circRNA inhibited RAFLS apoptotic rate. The Bcl-2 protein family is important to the apoptosis system and regulates the mitochondrial membrane permeability, including proapoptotic protein such as BAX and anti-apoptotic protein such as BCL-2 [18]. Herein, the study detected BAX and BCL-2 expression after circ_0002984 knockdown or overexpression and found that circ_0002984 increased BCL-2 protein production and decreased BAX protein expression, which supported the anti-apoptotic role of circ_0002984 in RAFLSs. Thus, these results indicated that circ_0002984 might promote RA development.

Evidencing evidence suggests that circRNA participates in multiple disease progression through combination with miRNAs, such as circ_0084615/miR-599 axis in colorectal cancer [19] and circFAM120A/miR-671-5p axis in RA [20]. The present work identified that circ_0002984 bound to miR-543 through mechanism assays. Functional research has demonstrated that miR-543 participates in biological processes as well as molecular functions in cancerous and noncancerous diseases, such as diabetic retinopathy, myelofibrosis and ovarian cancer [21, 22]. In addition, previous work reported that miR-543 induced FLS apoptosis through the plasmacytoma variant translocation 1/miR-543/SCUBE2 axis [23]. In this study, we also found that miR-543 was downregulated in synovial tissues of RA rats. Similarly, our data confirmed the downregulation of miR-543 in synovial tissues from RA patients and RAFLSs and the promoting effect of miR-543 in FLS apoptosis. Beyond that, we reported that miR-543 could inhibit FLS proliferation, migration, and TNF-α and IL-1β secretion. Importantly, circ_0002984-mediated regulation of FLS processes involved miR-543. Therefore, the circ_0002984/miR-543 pathway mediated FLS process.

Proprotein convertase subtilisins/kexins (PCSKs) are kinds of enzymes that can cleave immature target proteins and modulate the activity of metalloproteinases (MMPs) and cytokines [24]. PCSK6, as a member of PCSKs, participates in cell invasiveness, cytokine production and MMP activation [25, 26]. As reported, PCSK6 is a key protease in vascular remodeling, and the mechanism is associated with its regulation to contractile markers and MMP14 activation [27]. Additionally, this protein is involved in the development of some diseases, such as preeclampsia [28] and melanoma [29]. In particular, Wang et al. reported that PCSK6 silencing decreased proliferation and motility of RA synovial fibroblasts [30], indicating that PCSK6 knockdown might have a protective role in RA development. We identified that miR-543 targeted PCSK6, and PCSK6 expression was increased in synovial tissues from RA patients and RAFLSs. Moreover, circ_0002984-mediated regulation in RAFLSs involved PCSK6. As indicated by Jiang et al. PCSK6 can regulate RAFLS phenotypes by activating NF-κB, STAT3 as well as ERK1/2 pathways [31]. Thus, circ_0002984 could mediate the change of RAFLS phenotypes by the miR-543/PCSK6 axis.

However, further assay, particularly collagen-induced arthritis (CIA) mice assay, should be performed to analyze the effects of the circ_0002984/miR-543/PCSK6 axis on RA development in vivo. In addition to the miR-543/PCSK6 axis, other circ_0002984-regulated pathways may be involved in the regulation of circ_0002984 toward RAFLS processes. Further studies should be conducted to comprehensively assess circ_0002984-mediated RA development.

Taken together, circ_0002984 could regulate RAFLS proliferation, migration, inflammatory response and apoptosis through the miR-543/PCSK6 axis. These results suggested that circ_0002984 might contribute to RA development. Interfering circ_0002984 might be a therapeutic strategy for RA.

## Data Availability

The datasets used and analyzed during the current study are available from the corresponding author on reasonable request.
